# Effects of the Native Language on the Learning of Fundamental Frequency in Second-Language Speech Segmentation

**DOI:** 10.3389/fpsyg.2016.00985

**Published:** 2016-06-29

**Authors:** Annie Tremblay, Mirjam Broersma, Caitlin E. Coughlin, Jiyoun Choi

**Affiliations:** ^1^University of Kansas, LawrenceKS, USA; ^2^Radboud University NijmegenNijmegen, Netherlands; ^3^Hanyang UniversitySeoul, South Korea

**Keywords:** second language, speech segmentation, prosody, eye tracking, French

## Abstract

This study investigates whether the learning of prosodic cues to word boundaries in speech segmentation is more difficult if the native and second/foreign languages (L1 and L2) have similar (though non-identical) prosodies than if they have markedly different prosodies (Prosodic-Learning Interference Hypothesis). It does so by comparing French, Korean, and English listeners’ use of fundamental-frequency (F0) rise as a cue to word-final boundaries in French. F0 rise signals phrase-final boundaries in French and Korean but word-initial boundaries in English. Korean-speaking and English-speaking L2 learners of French, who were matched in their French proficiency and French experience, and native French listeners completed a visual-world eye-tracking experiment in which they recognized words whose final boundary was or was not cued by an increase in F0. The results showed that Korean listeners had greater difficulty using F0 rise as a cue to word-final boundaries in French than French and English listeners. This suggests that L1–L2 prosodic similarity can make the learning of an L2 segmentation cue difficult, in line with the proposed Prosodic-Learning Interference Hypothesis. We consider mechanisms that may underlie this difficulty and discuss the implications of our findings for understanding listeners’ phonological encoding of L2 words.

## Introduction

The segmentation of continuous speech into individual words is a particularly challenging task for non-native listeners, in that cues to word boundaries differ across languages. The cues that may be useful for segmenting the native language (L1) are often inefficient or even misleading for segmenting a second/foreign language (L2). Whether or not non-native listeners can learn to use segmentation cues has been shown to depend in part on the similarity between the L1 and the L2 (e.g., Weber and Cutler, 2006; Al-jasser, 2008; Tremblay et al., 2012; Tremblay and Spinelli, 2014). Unclear, however, is *how* L2 learning is shaped by the degree of similarity between the L1 and the L2. Most existing L2 speech segmentation studies have focused on L1–L2 pairings that differed drastically in how segmentation cues signal word boundaries (e.g., French–English, Japanese–English; Cutler et al., 1992; Cutler and Otake, 1994; Tremblay et al., 2012). It remains to be determined whether segmentation cues such as prosody are more difficult to learn if the L1 and L2 prosodies pattern in non-identical but similar ways (henceforth, ‘similar[ly]’) in how they signal word boundaries than if they are drastically different. Assessing whether L1–L2 similarity hurts the learning of L2 segmentation cues may in turn shed important light on the cognitive mechanisms that underlie such learning and on L2 learners’ phonological encoding of L2 words.

The present study tests whether the learning of a new segmentation cue is more difficult if the L1 and L2 prosodic systems are similar than if they are markedly different. We will refer to this as the *Prosodic-Learning Interference Hypothesis*. For this hypothesis, similarity is operationalized as a given prosodic cue (e.g., fundamental frequency [F0] rise) signaling the *same word boundary* in both the L1 and the L2 (e.g., F0 rise signals word-final boundaries in both languages). For learning to take place, the L1 and L2 prosodic systems need by definition not to be identical. Hence, the L1 and L2 prosodic systems will be considered similar, though not identical, if a given prosodic cue signals the same word boundary in the L1 and L2 prosodic systems but does so differently (e.g., the alignment of the word-final F0 rise differs between the L1 and the L2). In contrast, the L1 and L2 prosodic systems will be considered different if a given prosodic cue signals *different word boundaries* in the L1 and the L2 (e.g., F0 rise signals word-initial boundaries in the L1 but word-final boundaries in the L2).

Upon initial inspection, the existing literature on non-native speech segmentation appears to suggest that the use of L1 cues is beneficial to L2 speech segmentation when the L1 and L2 pattern similarly. For example, Murty et al. (2007) have shown that listeners whose L1 is Telugu, a Dravidian language that resembles Japanese in its mora-timed rhythm, segment Japanese words similarly to native Japanese listeners, whereas listeners from non-mora-timed L1s (French and English) had not been found to do so (Otake et al., 1993; Cutler and Otake, 1994). Similarly, Kim et al. (2008) have found that listeners whose L1 is Korean, a syllable-timed language, segment French words similarly to native French listeners, whereas listeners from non-syllable-timed L1s (English, Dutch, and Japanese) had not been found to do so (Cutler et al., 1983, 1986; Otake et al., 1996; Cutler, 1997). However, given the difficulty in quantifying rhythmic similarity across languages, the actual degree of similarity between Telugu and Japanese and between Korean and French remains unclear.^1^

Prosody, specifically F0 information, may provide a better test case for assessing how the learning of L2 segmentation cues is shaped by the degree of similarity between the L1 and the L2, in that F0 can be measured relatively independently of the segmental content of languages, thus facilitating direct comparisons across languages.^2^ There are good reasons to hypothesize that the learning of F0 cues may be more difficult if the L1 and L2 prosodic systems are similar than if they are completely different. First, L2 learners may perceive the F0 movement in the L1 and the L2 as identical and thus not readjust their use of segmentation cues. This perceptual assimilation would be similar *in spirit* to Best and Tyler’s (2007) Perceptual Assimilation Model of L2 speech perception (PAM-L2; see also Best, 1995) and to Flege’s (1995) Speech Learning Model (SLM), where L2 learners do not accurately perceive or produce L2 phonemes as a result of assimilation to L1 phonemes. Second, L2 learners may only readjust their use of F0 cues if these unadjusted cues do result in parsing errors, namely in the greater activation of L2 competitor words over L2 target words. In other words, parsing failure may be necessary to trigger L2 learning (for such a proposal, see Carroll, 2004).

The present study tests the Prosodic-Learning Interference Hypothesis by examining how Korean- and English-speaking L2 learners of French use F0 rise to locate phrase-final (thus, also word-final) boundaries in French.^3^ In French, the last non-reduced syllable of the last content word of the accentual phrase (AP) receives a pitch accent in non-utterance-final position, and the first or second syllable of the first content word in the AP can optionally receive a phrase accent (e.g., Jun and Fougeron, 2000, 2002; Welby, 2006). For example, in [*un gentil chaton*]_AP_ ‘a nice kitty,’ a phrase accent can be aligned with the first syllable of *gentil* and a pitch accent is aligned with the last syllable of *chaton*. The basic underlying tonal pattern of the AP in French is L(HL)H^∗^, where H represents a high phrase accent, H^∗^ represents a high pitch accent, and L represents low tones (e.g., Jun and Fougeron, 2002; Welby, 2006). The predominant acoustic cues to (non-utterance-final) pitch accents in French are a rise in F0 and lengthening, whereas the predominant cue to phrase accents is an F0 rise (Welby, 2006). Whereas the F0 in pitch accents rises until the end of the AP-final syllable, the F0 in phrase accents is usually lower, flatter, and more variable in its slope and alignment earlier in the AP. Lengthening and F0 rise aligned with the right edge of the AP-final syllable are thus reliable cues to word-final boundaries in AP-final position in French, whereas a flatter F0 rise earlier in the AP can cue word-initial boundaries (e.g., Christophe et al., 2004; Bagou and Frauenfelder, 2006; Welby, 2007; Spinelli et al., 2007, 2010).

Previous studies have shown that native French listeners locate word-final boundaries at the offset of both lengthened syllables (e.g., Banel and Bacri, 1994; Bagou et al., 2002) and syllables with an F0 rise (e.g., Bagou et al., 2002; Bagou and Frauenfelder, 2006). Christophe et al. (2004) provided further evidence that phrase-final prosodic boundaries (and pitch accents) mediate lexical access in French. They found that monosyllabic words (e.g., *chat* [ʃa] ‘cat’) were recognized more slowly when they were temporarily ambiguous with a competitor word created segmentally between the monosyllabic word and the first syllable of the following word (e.g., *chagrin* [ʃagʁ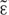] ‘heartache’ in *[d’un chat grincheux]_AP_* [d
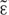
ʃagʁ
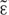
ʃø] ‘of a cranky cat’) than when they were not temporarily ambiguous with such a competitor (e.g., *[d’un chat drogué]_AP_* [d
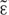
ʃadʁoge] ‘of a drugged cat’; [ʃadʁo] is not a French word); however, if the monosyllabic word was at an AP-final boundary and thus received a pitch accent (e.g., *[le gros chat]_AP_ [grimpait aux arbres]_AP_* [lǝgʁoʃa gʁ
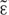
pεozaʁbʁ] ‘the big cat was climbing trees’), the target word was no longer recognized more slowly when it was temporarily ambiguous with a phonemic competitor than when it was not (e.g., *[le gros chat]_AP_ [dressait l’oreille]_AP_* [lǝgʁεsεloʁεj] ‘the big cat was sticking up his ears’; [ʃadʁε] is not a French word). These findings suggest that phrase-final boundaries, marked with a pitch accent and thus realized with both lengthening and an F0 rise, act as filter and constrain lexical access (see also Michelas and D’Imperio, 2010). In an artificial-language segmentation study, Tyler and Cutler (2009) also showed that French listeners independently use F0 and duration cues to word-final boundaries.

Korean is similar to French in that prominence is also at the level of the AP. In the Seoul dialect, the basic underlying tonal pattern of the AP is (LH)LH or (HH)LH, with the first tone being H if the first sound is tense or aspirated and L otherwise (e.g., Jun, 1995, 1998, 2000; Beckman and Jun, 1996). For example, in [jǝ
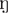
man-ine-n
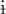
n]_AP_ ‘youngman-family-topic,’ the first H is “loosely aligned” with the second syllable of the phrase and the second H is aligned with the final syllable of the phrase (Jun, 1998, pp. 195, 196). Thus, like French, Korean has an H tone on the AP-final (and thus word-final) syllable, which can cue word-final boundaries in that AP-final position. However, unlike French, the phrase-final F0 rise in Korean peaks before the syllable offset and begins decreasing thereafter such that it is already low in the next syllable, whereas in French the F0 begins decreasing *after* the accented syllable (cf. Jun, 2000, p. 21, vs. Jun and Fougeron, 2002, p. 163). Korean also differs from French in that lengthening does not consistently cue AP-final boundaries in Korean (cf. Oh, 1998 and Cho and Keating, 2001, vs. Jun, 1993; Chung et al., 1996); however, syllables at the end of the intonational phrase (IP) are consistently lengthened in both Korean (e.g., Jun, 1993, 1995, 1998, 2000; Cho and Keating, 2001) and French (e.g., Jun and Fougeron, 2000, 2002). In that sense, French and Korean are similar but not identical in how they cue word-final boundaries.

Like French listeners, Korean listeners use prosodic cues to phrase-final accents for locating word-final boundaries in continuous speech. In an artificial-language segmentation study, Kim et al. (2012) showed that Korean listeners use both F0 and lengthening as cues to word-final boundaries. Similarly, in word-spotting experiments, Kim and Cho (2009) demonstrated that Korean listeners recognized (prototypical) LH words more easily when these words were preceded by a syllable containing an H tone than when they were preceded by an L tone; however, the same was not true of (atypical) HL words that were preceded by a syllable containing an L tone. Kim and Cho (2009) further showed that the L tone at the onset of the target disyllabic words was not helpful for segmentation if it was not preceded by an H tone, suggesting that it is the contrast in F0 tones that enhances Korean listeners’ segmentation of Korean speech, but only if H is in word-final position. Kim and Cho (2009) also showed that Korean listeners benefited from lengthening at least under some circumstances.^4^

English differs from both French and Korean in that prominence is lexical rather than phrasal, and pitch accents are aligned with stressed syllables and they are not necessarily phrase-final (e.g., Beckman and Elam, 1997). Statistically, stress tends to be word-initial rather than word-final, especially in nouns (e.g., Cutler and Carter, 1987; Clopper, 2002). Stress in accented words thus provides a somewhat reliable cue to word-initial boundaries in English (e.g., Cutler and Butterfield, 1992; McQueen et al., 1994; Mattys, 2004). The primary prosodic correlates of stressed syllables in accented English words are F0 rise, increased duration, and greater intensity (e.g., Lieberman, 1960; Beckman, 1986), and the importance of each depends in part on the location of the accented syllable in the word (e.g., Tremblay and Owens, 2010) and on the location of the word in the phrase (e.g., Tyler and Cutler, 2009). It is thus the case that English is quite different from French in how prosodic cues signal word boundaries.

English listeners tend to parse accented syllables as word-initial. This was shown in a variety of experimental paradigms (e.g., juncture perception task: Cutler and Butterfield, 1992; word-spotting tasks: McQueen et al., 1994; cross-modal priming tasks: Mattys, 2004; Mattys et al., 2005). However, because stress is strongly correlated with vowel quality in English, English listeners make limited use of prosodic cues to stress in the absence of segmental cues to stress (e.g., Cutler and Clifton, 1984; Cutler, 1986; Small et al., 1988; Fear et al., 1995; Cooper et al., 2002). When English listeners do use prosodic cues to word boundaries, they associate F0 rise with word-initial boundaries (Tyler and Cutler, 2009). This is indeed what we should expect given the statistical tendency for stress to occur word-initially. Interestingly, English listeners also appear to associate lengthening to word-final boundaries (Tyler and Cutler, 2009), suggesting that different prosodic cues can signal different word boundaries in English. Tyler and Cutler (2009) attribute the facilitative effect of word-final lengthening to the phrase- (and thus word-) final lengthening that occurs in English and many other languages (see also Vaissière, 1983; Hayes, 1995).

The similarities and differences among French, Korean, and English allow us to test whether the learning of a new segmentation cue is more difficult if the L1 and L2 prosodic systems are similar than if they are markedly different. French and Korean pattern similarly in that their prosody is phrasal, and for words in AP-final position, word-final boundaries are cued by an F0 rise; yet, they differ in that the AP-final F0 peak is aligned differently in the two languages (earlier in Korean, later in French). In contrast, English differs from both French and Korean in that prominence is lexical and F0 rise signals word-initial rather than word-final boundaries. If the learning of a new segmentation cue is more difficult when the L1 and L2 prosodic systems are similar than when they are different, Korean L2 learners of French should have more difficulty in using F0 cues to word-final boundaries in French than *both* native French listeners and English L2 learners of French.

In a word-monitoring experiment, Tremblay et al. (2012) examined French and English listeners’ use of F0 and duration cues to word-final boundaries in French. In an adaptation of Christophe et al. (2004), they asked native French listeners and mid- and high-proficiency English L2 learners of French to monitor disyllabic words that were not in the stimuli but that were created phonemically between a monosyllabic noun and the first syllable of the following word (e.g., *chalet* ‘cabin’ in *chat lépreux* ‘grumpy cat’). In the across-AP condition, the monosyllabic word in the stimuli (e.g., *chat*) received a pitch accent, and thus the disyllabic word to be monitored (e.g., *chalet*) crossed an AP boundary (e.g., *[[Le chat]_AP_ [lépreux et légendaire]_AP_]_PP_ s’endort doucement* ‘The leprous and legendary cat is slowly falling asleep’); in the within-AP condition, the monosyllabic word in the stimuli (e.g., *chat*) was not accented, and thus the disyllabic word to be monitored (e.g., *chalet*) was located within an AP (e.g., *[[Le chat lépreux]_AP_]_PP_ s’endort doucement* ‘The leprous cat is slowly falling asleep’). If prosody constrained lexical access, participants should show fewer detections of the disyllabic word to be monitored (i.e., fewer false alarms) in the across-AP condition than in the within-AP condition. Experiment 1 used natural stimuli; in Experiment 2, stimuli were resynthesized such that the F0 was swapped between the across-AP and within-AP conditions, thus making it possible to examine the effect of F0 cues independently of duration cues. Different participants at similar proficiencies completed Experiments 1 and 2.

The results of Experiment 1 showed that the high-level L2 learners and native listeners, but not the mid-level L2 learners, had fewer false alarms in the across-AP condition than in the within-AP condition, indicating that sufficiently advanced English L2 learners of French could parse accented syllables as word-final. However, the results of Experiment 2 showed that only the native listeners were able to use F0 cues to word-final boundaries. These results suggest that unlike French listeners, English listeners were not able to use F0 rises as a cue to word-final boundaries in French; they could only use duration as a cue to word-final boundaries, but only if they were sufficiently proficient in French (for details, see Tremblay et al., 2012).

The present study uses the same stimuli as those used in Experiment 2 of Tremblay et al. (2012), but in a visual-world eye-tracking experiment, thus shedding light on the time course of activation of target and competitor words as listeners hear F0 cues to word-final boundaries. We examine the segmentation of French speech by native French listeners and by *both* English and Korean L2 learners of French, with the L2 listeners being matched not only in their French proficiency, but also in *all* their language background information. Thus, if any difference is found between the L2 groups, such a difference could only be attributed to the participants’ L1. The use of eye tracking will allow us to determine not only if Korean L2 learners of French have more difficulty than English L2 learners of French in using F0 cues to word-final boundaries in French, but also if English L2 learners of French can in fact learn to use F0 cues to word-final boundaries in French, something that was not found in Tremblay et al. (2012).

## Materials and Methods

### Ethics Statement

The study was approved by the Human Subjects Committee of the University of Kansas, Lawrence. Participants read and signed a written consent form. No vulnerable population was involved.

### Participants

Twenty-five native French listeners (mean age: 26.4, SD: 4.6), 16 English L2 learners of French (mean age: 23.9, SD: 0.9), and 16 Korean L2 learners of French (mean age: 23.3, SD: 8.2) participated in this study. The English listeners were undergraduate or graduate students at a Midwestern university in the US who either majored in French or identified themselves as having functional proficiency in French. The Korean listeners were undergraduate students majoring in French or in French-Korean translation at a university in Seoul, Korea.^5^ All participants had normal or corrected-to-normal vision, and no participants reported any hearing impairment. All participants received monetary compensation or course credit in exchange for their time.

The L2 learners filled out a language background questionnaire and completed a cloze test that would assess their proficiency in French (Tremblay, 2011). Their language background information and proficiency scores are summarized in **Table 1.** The English and Korean listeners were matched in both their experience with French and their proficiency in French.^6^ One-way ANOVAs with L1 as between-group variable did not reveal significant differences between the two groups on any of the language background variables or on the proficiency scores (*p* > 0.1).

**Table 1:** L2 learners’ language background information and proficiency scores.

	AFE^a^	YrsInstr^b^	MthsRes^c^	%Use^d^	Cloze
English L2 learners of French (*n* = 16)	16.8 (4.3)	6.2 (3.2)	14.0 (23.5)	13.7 (10.2)	23.3 (8.2)
Korean L2 learners of French (*n* = 16)	18.8 (2.1)	5.7 (2.2)	14.3 (15.8)	12.5 (10.6)	21.7 (5.7)


*Mean (standard deviation). ^a^Age of first exposure to French. ^b^Number of years of formal instruction on French. ^c^Months of residence in a French-speaking environment. ^d^Percent weekly use of French.*

All Korean listeners also had some knowledge of English. On a scale from 1 to 4 (1 = beginner, 2 = intermediate, 3 = advanced, 4 = near-native), they rated their English-listening skills as similar to their French-listening skills (English: mean: 2.6, SD: 0.7; French: mean: 2.4, SD: 0.7; *t* < |1|).

### Materials

All stimuli came from Tremblay et al. (2012). Participants heard sentences in which a competitor word was created segmentally between a monosyllabic target word and the first syllable of the disyllabic adjective following it (e.g., *chalet* ‘cabin’ in *chat lépreux* ‘leprous cat’). In the across-AP condition, the monosyllabic target word received a pitch accent, and the disyllabic competitor word crossed an AP boundary (e.g., *[[Le chat]_AP_ [lépreux et légendaire]_AP_]_PP_ s’endort doucement* ‘The leprous and legendary cat is slowly falling asleep’). The first AP contained an LH^∗^ tonal pattern, with the L tone belonging to either a phrase-initial accent or a pitch accent and the H^∗^ tone belonging to a pitch accent. In the within-AP condition, the pitch accent instead fell on the last syllable of the post-nominal adjective (e.g., *[[Le chat lépreux]_AP_]_PP_ s’endort doucement* ‘The leprous cat is slowly falling asleep’). The AP in this condition contained an LLH^∗^ tonal pattern, with the first L tone belonging to a phrase-initial accent and the LH^∗^ tones belonging to a pitch accent.

The auditory stimuli were recorded by a female native speaker of French from Bordeaux (France) using a Marantz PMD 750 solid-state recorder and head-mounted condenser microphone. The speaker was trained to produce the stimuli such that an H^∗^ tone would fall on the monosyllabic noun in the across-AP condition but on the last syllable of the post-nominal adjective in the within-AP and control conditions. In both experimental conditions, the peak F0 of the H^∗^ tone was aligned with the AP-final boundary. The H^∗^ tone produced on the monosyllabic noun in the across-AP condition was not followed by a pause so that the disyllabic competitor word could be erroneously detected.

Next, the F0 contours of the across-AP and within-AP conditions were resynthesized such that the F0 of the first four syllables was swapped between the two experimental conditions. The first four syllables of the resynthesized across-AP sentences thus contained the F0 contour of the corresponding syllables in the within-AP condition, and the first four syllables of the resynthesized within-AP sentences contained the F0 contour of the corresponding syllables in the across-AP condition. This manipulation, which made it possible to examine the effect of F0 rise independently of duration, resulted in four conditions: (i) an across-AP condition with F0 rise (natural); (ii) an across-AP condition without F0 rise (F0 rise removed); (iii), a within-AP condition with F0 rise (F0 rise added); and (iv) a within-AP condition without F0 rise (natural).

**FIGURE 1:**
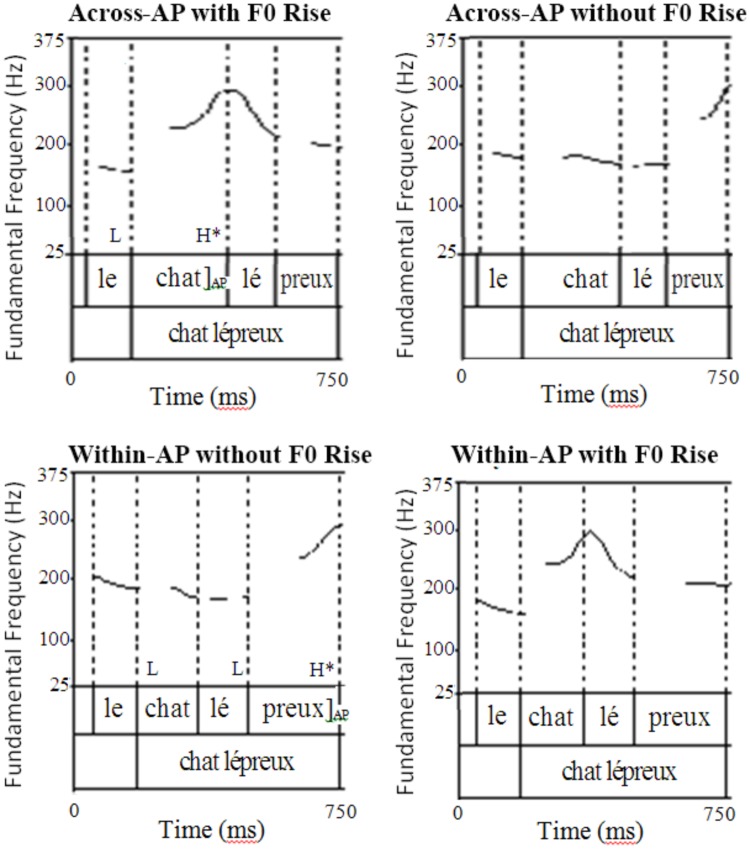
**Example of natural and resynthesized stimuli (adapted from Figure 4 of Tremblay et al., 2012)**.

The experimental stimuli were resynthesized using close-copy stylization (e.g., de Pijper, 1983). The first four syllables of the experimental items were divided into 20 segments each, and the average F0 of each segment was extracted. The existing pitch points in each segment were then dragged vertically using the Pitch Synchronous OverLap-Add (PSOLA) method in Praat (Boersma and Weenink, 2004) so that they would approximate the value of the extracted average in the corresponding segment of the opposite condition. After the initial resynthesis, the pitch contour of the natural and resynthesized conditions were closely examined, and resynthesized contours that were judged not to be sufficiently similar to the natural contours of the opposite condition were altered so that they would better approximate them. Once the contours were judged to be satisfactory, a stop Hann-band filter from 500 to 1,000 Hz with a smoothing of 100 Hz was applied to all the stimuli to mask the occasionally robotic sound that resulted from the F0 manipulation. This filter did not significantly affect the segmental quality of the stimuli. **Figure 1** shows an example of natural and resynthesized stimulus in the across-AP and within-AP conditions (adapted from Figure 4 of Tremblay et al., 2012).

Acoustic analyses of the first two syllables in the stimuli (e.g., *le chat*) performed in Praat (Boersma and Weenink, 2004) are reported in Tremblay et al. (2012). In brief, these analyses revealed that the prosodic cue manipulation was successful, with the resynthesized monosyllabic word (e.g., *chat*) having a significantly different F0 in the across-AP and within-AP sentences.

The experiment included a total of 32 experimental stimuli randomly interspersed with the 69 filler stimuli, 8 of which were used in the practice session. The participants were assigned to one of four lists and saw each experimental item in only one condition (total: 8 items per condition; for the complete list of experimental items, see Tremblay et al., 2012).

Participants saw four words on the computer display and clicked on the word they thought they heard. In the experimental stimuli, the display included the target (monosyllabic) word (e.g., *chat*), the competitor (disyllabic) word (e.g., *chalet*), and two distracter words. To ensure that the participants would not be biased in their fixations toward the target and competitor words (given their segmental overlap), the distracter words also overlapped together in their segmental content. These distracter words were either both monosyllabic (e.g., *clé* ‘key’ and *craie* ‘chalk’; 6 items), both disyllabic (e.g., *chemin* ‘path’ and *cheval* ‘horse’; 6 items), or one of each (e.g., *prince* ‘prince’ and *principe* ‘principle’; 20 items), and they did not overlap segmentally or semantically with the target and competitor words. Since the words across the four prosodic conditions are identical, L2 learners’ familiarity with the words in the display cannot explain any prosodic effect that we may find (for discussion, see Tremblay et al., 2012).

All words in the visual display were presented orthographically (for a validation of this method, see Huettig and McQueen, 2007; McQueen and Viebahn, 2007). It was decided to present the words orthographically rather than with images, first because not all the experimental words were easily imageable, and second to facilitate the task with L2 learners, who may not have equal familiarity with all the words in the experiment. Since prosody is independent of word spelling in French, this characteristic of our experimental design does not pose any concern.

### Procedures

The eye-tracking experiment was designed and compiled with Experiment Builder software (SR Research), and the participants’ eye movements were recorded with an Eyelink eye tracker (SR Research) at a sampling rate of either 250 Hz or 1,000 Hz, depending on the location of the data collection. An ASIO-compatible sound card was used on the display computer to ensure that the audio timing would be accurate.

The experiment began with a calibration of the eye tracker using the participants’ right eye. If the eye tracker could not be successfully calibrated with the participant’s right eye, his/her left eye was instead used. This initial calibration was followed by a practice session (8 trials) and by the main experiment (93 trials). In each trial, the participants saw four orthographic words in a (non-displayed) 2 × 2 grid for 4,000 ms. The words then disappeared and a fixation cross appeared in the middle of the screen for 500 ms. As the fixation cross disappeared, the four words reappeared on the screen in their original position and the auditory stimulus was heard (synchronously) over headphones. The participants were instructed to click on the target word with the mouse as soon as they heard the target word in the stimulus. The participants’ eye movements were measured from the onset of the target word (e.g., the onset of *chat*). The trial ended with the participants’ response, with an inter-trial interval of 1,000 ms.

The 32 experimental and 61 filler trials were pseudo-randomized and presented in four blocks (23 trials per block, except for one block that contained 24 trials). Each block contained 8 experimental trials (2 from each condition). Both the order of the experimental and filler trials within a block and the order of blocks were randomized across participants. The participants took a break after completing the second block. The eye tracker was calibrated at the beginning of each block and whenever it was necessary during the experiment. The participants completed the experiment in approximately 15–20 minutes.

### Data Analysis and Predictions

Experimental trials that received distracter responses (rather than target or competitor responses) or for which eye movements could not reliably be tracked were excluded from the analyses. This resulted in the exclusion of 6.4% of all trials (2.7% for French listeners, 1.5% for Korean listeners, and 2.2% for English listeners). For the remaining trials, we analyzed the participants’ eye movements in each of the four regions of interest (corresponding to the four orthographic words on the screen).

Proportions of fixations to the target, competitor, and distracter words were extracted in 8-ms time windows from the onset of the target word to 1,500 ms after the target word. To better capture any effect of lexical competition due to the manipulated F0 cues, statistical analyses were conducted on the *difference* between target and competitor fixations (i.e., competitor fixations were subtracted from target fixations). This difference factors out any difference in the speed with which participants begin to fixate both target and competitor words (over distracter words), thus making the data more comparable between native listeners and L2 learners.

Listeners’ fixation differences were modeled using growth curve analysis (GCA; Mirman, 2014). GCAs are similar to mixed-effects models (for discussion, see Baayen, 2008), but they also include time coefficients, thus enabling researchers to model participants’ fixations over time. GCA is ideal for analyzing participants’ proportions of fixations as the speech signal unfolds, because they can model cross-over effects in fixations that cannot always be captured in traditional time-window analyses of eye-tracking data. For example, if Fixation Line A is 10% higher than Fixation Line B from 200 to 300 ms but 10% lower than Fixation Line B from 400 to 500 ms (with the two lines intersecting at 350 ms), a time window analysis that averages fixations from 200 to 500 ms would likely show no difference between the two lines, when in fact the directionality of the effect evidenced by the two lines reversed half way through the time window. GCAs can thus model subtle changes in the curvilinear patterns of eye fixations over time, capturing differences in the slope and curvature of the fixation lines. GCAs also have the advantage of not requiring (potentially arbitrary) decisions regarding the critical time window for the statistical analysis.

GCAs include orthogonal time coefficients, the fixed variables of interest, and random variables. The time coefficients model the shape of the proportions of fixations over time. In a visual-world eye-tracking paradigm, the difference between participants’ target and competitor fixations typically takes the form of an ‘s’-shaped (i.e., cubic) line, with fixations initially being flat (and sometimes decreasing depending on the degree of competition), then increasing in a steady slope, and finally leveling off. The analysis in this study thus includes linear, quadratic, and cubic time coefficients. The time coefficients are centered, and they are made orthogonal prior to entering them in the analyses because these time coefficients would otherwise be highly correlated, which would make the model unstable and the results difficult to interpret. The fixed variables in GCAs are those of the experimental design.

The results of GCAs are interpreted as follows: For the researcher to be able to conclude that a manipulation of the speech signal resulting in two different conditions has an effect on participants’ fixations, the GCA must show an *interaction* between this manipulation and at least one of the time coefficients. Such an interaction indicates that as the speech signal unfolds over time, the shape of participants’ fixation line changes differently for the two conditions. Finding only an effect of experimental variable and no interaction between it and any of the time coefficients indicates that fixation proportions are higher or lower in one condition than in another, but the shape of participants’ fixation lines is similar across the two conditions. Hence, such an effect could not be attributed to any manipulation of the speech signal (i.e., such an effect would be better interpreted as a baseline effect). The GCAs in the present study included two fixed variables: whether or not the word-final boundary was signaled by an F0 rise (within-participant), and the native language of the participants (between-participant), with native French listeners being compared to English and Korean L2 learners of French in a first analysis and with the L2 groups being compared to each other in a second analysis. Because the test items in the across-AP and within-AP conditions differ in their duration, they cannot be compared directly in a GCA analysis of listeners’ proportions of fixations. Hence, we examined the effects of F0 rise and L1 separately for the across-AP and within-AP conditions.

Like mixed-effects models, GCAs can also include crossed random variables. The GCAs proposed by Mirman (2014) include participant as random intercept and the orthogonal time coefficients as random slopes for the participant variable, thus allowing the analysis to model a line of a different shape for each participant. Such an analysis is ideal to capture between-participant variability in their fixations over time.^7^

The GCAs were run using the lme4 package in R (Bates et al., 2015). The initial analysis included the three time coefficients, the presence or absence of a word-final F0 rise, listeners’ L1, and all interactions as fixed effects, and it included participant as random intercept and the three time coefficients as random slope for the participant variable. The fixed effects other than the three time coefficients were then removed from the model one at a time, and model comparisons were run in pairwise fashion to determine if the more complex model accounted for significantly more of the variance, as determined by log-likelihood ratio tests. We report the simplest model including the three time coefficients that accounted for significantly more of the variance than simpler models. If the best model yielded significant interactions involving L1, follow-up GCAs were conducted separately for each of the L1. The alpha level of these subsequent models was adjusted manually using the Bonferroni correction.

If the presence of a word-final F0 rise enhances speech segmentation, the GCAs should yield both an effect of F0 rise (with larger difference between target and competitor fixations in the condition with an F0 rise than in the condition without such a rise) and an interaction between this F0 rise and at least one of the three time coefficients, indicating that the differential fixation lines in the condition with vs. without an F0 rise have different shapes as the speech signal unfolds. If participants’ L1 modulates their ability to use this F0 rise, the GCAs should yield three-way interactions between the presence and absence of a word-final F0 rise, participants’ L1, and at least one of the time coefficients.

## Results

French, English, and Korean listeners’ proportions of target, competitor, and distracter fixations in the across-AP and within-AP conditions are presented in the figures in Appendix A in the supplemental data.

### Across-AP Condition

#### All Listeners

**FIGURE 2:**
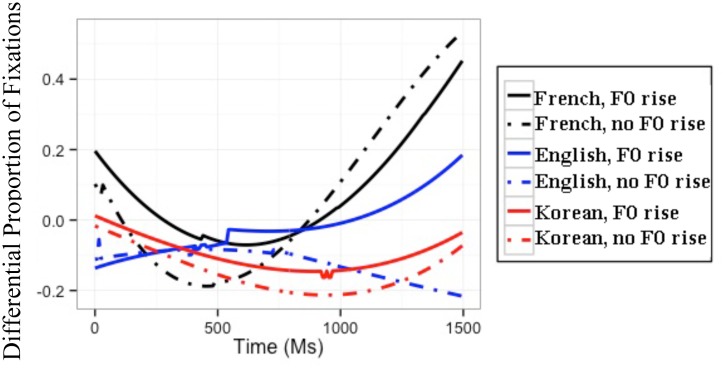
**Modeled difference between proportions of target and competitor fixations for the three L1 groups in the across-AP condition.** The difference is calculated as the fixations to targets minus those to competitors; hence, positive values reflect more fixations to targets than to competitors.

Recall that the across-AP condition naturally contained an F0 rise, and F0 was resynthesized such that it would be flat. The best GCA on the difference between listeners’ proportions of target and competitor fixations in the across-AP condition included all simple effects and all interactions. The results of this GCA and the interpretation of the GCA coefficients can be found in Appendix B in the **supplemental data (Table B1**). The modeled differences between target and competitor fixations (henceforth, differential fixations) are illustrated in **Figure 2.**

Among other effects, the GCA yielded significant three-way interactions between F0, L1, and the time coefficients. In order to understand the directionality of these three-way interactions, subsequent GCAs were performed on the differential proportions of fixations separately for each L1 group. For French and English listeners’ differential fixations, these subsequent GCAs with all simple effects and all interactions had the best fit. For Korean listeners’ differential fixations, the best GCA included F0 and the time coefficients, but no interaction between them. The results of these subsequent GCAs are presented in **Table 2.** For each group, the baseline is the difference between the proportions of target and competitor fixations in the condition *with* an F0 rise (i.e., the natural speech condition). Because the time coefficients were made orthogonal, any effect of a fixed variable (e.g., F0, L1) is to be interpreted on the averaged differential fixations over time (Mirman, 2014).

**Table 2:** Growth curve analyses on the difference between listeners’ target and competitor fixations in the across-AP condition separately for French, English, and Korean listeners.

Group	Variable	Estimate (SE)	*t*
French	(Intercept)	0.094	2.566
	
	Time		
	Linear	0.783	1.513
	Quadratic	1.856	3.912^∗∗^
	Cubic	-0.214	<|1|
	
	F0	-0.016	-2.580^∗^
	
	Time × F0		
	Linear	1.247	14.928^∗∗∗^
	Quadratic	0.202	2.432^∗^
	Cubic	-0.492	-5.891^∗∗∗^

English	(Intercept)	0.087	<|1|
	
	Time		
	Linear	-0.902	<|1|
	Quadratic	1.523	1.179
	Cubic	-0.252	<|1|
	
	F0	-0.099	-11.931^∗∗∗^
	
	Time × F0		
	Linear	-1.501	-13.211^∗∗∗^
	Quadratic	-0.566	-4.992^∗∗∗^
	Cubic	-0.116	-1.021

Korean	(Intercept)	-0.097	-3.486^∗∗^
	
	Time		
	Linear	-0.354	<|1|
	Quadratic	0.588	2.214
	Cubic	0.100	<|1|
	
	F0	-0.051	-7.482^∗∗∗^


*α = 0.0167;^∗^ = *p* < 0.0167;^∗∗^ = *p* < 0.003;^∗∗∗^ = *p* < 0.0003. French model: *n* = 25; 9,236 observations. English model: *n* = 16; 5,833; observations. Korean model: *n* = 16; 6,012 observations.*

#### French Listeners

For the GCA on French listeners’ data, the significant positive *t* value for the quadratic time coefficient indicates that French listeners’ differential fixation line in the condition with an F0 rise had a convex shape. The significant negative *t* value for F0 means that French listeners had a lower differential proportion of fixations in the condition without an F0 rise than in the condition with an F0 rise. Crucially, the significant positive *t* values for the interaction between F0 and the linear and quadratic time coefficients indicate that French listeners had a differential fixation line that had more of an ascending slope and was more convex in the condition without an F0 rise than in the condition with an F0 rise. Furthermore, the significant negative *t* value for the interaction between F0 and the cubic time coefficient means that the French listeners’ differential fixation line in the condition without an F0 rise had more of a canonical ‘s’ shape than their differential fixation line in the condition with an F0 rise.

These results can be observed in the modeled differential proportions of fixations in **Figure 2**: In the absence of an F0 rise, French listeners showed lower differential proportions of fixations, thus more lexical competition, during the first 750 ms *post* target-word onset, after which fixations became more similar between the two F0 conditions. The absence of an F0 rise thus modulates French listeners’ fixations early on in the word recognition process, making it more difficult to locate the word-final boundary and resulting in increased lexical competition.

#### English Listeners

For the GCA on English listeners’ data, the significant negative *t* value for F0 indicates that English listeners had a lower differential proportion of fixations in the condition without an F0 rise than in the condition with an F0 rise. Importantly, the significant negative *t* values for the interaction between F0 and the linear and quadratic time coefficients mean that English listeners’ differential fixation line had more of a descending slope and more of a concave shape in the condition without an F0 rise than in the condition with an F0 rise.

These results can also be seen in the modeled differential proportions of fixations in **Figure 2**: English listeners’ differential proportions of fixations in the two F0 conditions were similar up until 500 ms post target-word onset, after which English listeners showed lower differential proportions of fixations, thus more lexical competition, in the condition without an F0 rise than in the condition with an F0 rise. The absence of F0 rise thus modulates English listeners’ fixations later on in the word recognition process. In other words, English listeners could incorporate the use of F0 cues to word-final boundaries in the segmentation of French speech (unlike the results of Tremblay et al., 2012), but did so later than French listeners.

#### Korean Listeners

Finally, for the GCA on Korean listeners’ data, the significant negative *t* value for the intercept means that Korean listeners’ differential proportion of fixations in the condition with an F0 rise was lower than 0, and the significant negative *t* value for F0 indicates that Korean listeners had a lower differential proportion of fixations in the condition without an F0 rise than in the condition with an F0 rise. Importantly, the interaction between F0 and time did not make it to the model, indicating that Korean listeners’ use of F0 did not change as a function of time.

These results are illustrated in the modeled differential proportions of fixations in **Figure 2**: Although Korean listeners showed an effect of F0 in the predicted direction, this effect of F0 did not change as the speech signal unfolded. The effect of F0 can therefore not be attributed to Korean listeners’ intake of the speech signal.

#### L2 Listeners

To ascertain whether Korean listeners differed significantly from English listeners in their differential proportions of fixations, an additional GCA was run only on the L2 learners’ data in the across-AP condition, with the English group as baseline. The model with the best fit included all simple effects and all interactions. The results of this GCA and the interpretation of the GCA coefficients can be found in Appendix B in the **supplemental data (Table B2**). In brief, this GCA revealed significant three-way interactions between L1, F0, and the linear and quadratic time coefficients, indicating that English and Korean listeners differed from each other in the effect of F0 as a function of time: English listeners’ differential fixation lines for the two F0 conditions differed in their quadratic shape; Korean listeners did not show this effect (cf. **Table 2**).

### Within-AP Condition

#### All Listeners

**FIGURE 3:**
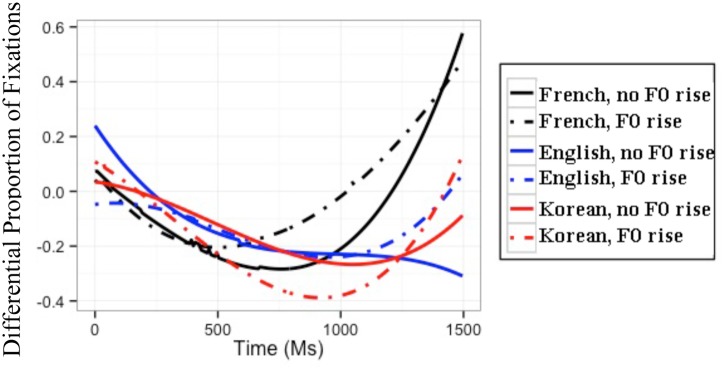
**Modeled difference between proportions of target and competitor fixations for the three L1 groups in the within-AP condition.** The difference is calculated as the fixations to targets minus those to competitors; hence, positive values reflect more fixations to targets than to competitors.

Recall that the within-AP condition naturally did *not* contain an F0 rise, and the flat F0 was resynthesized such that the target word would end with an F0 rise. The best GCA on the difference between listeners’ proportions of target and competitor fixations in the within-AP condition included all simple effects and all interactions. The results of this GCA and the interpretation of the GCA coefficients can be found in Appendix B in the **supplemental data (Table B3**). The modeled differences between target and competitor fixations are illustrated in **Figure 3.**

Among other effects, the GCA yielded significant three-way interactions between F0, L1, and the time coefficients. Again, in order to understand the directionality of these three-way interactions, subsequent GCAs were run on the differential proportions of fixations separately for each L1 group. For French, English, and Korean listeners’ differential fixations, these subsequent GCAs with all simple effects and all interactions had the best fit. The results of these subsequent GCAs are presented in **Table 3.** For each group, the baseline is the difference between the proportions of target and competitor fixations in the condition *without* an F0 rise (i.e., the natural speech condition). Again, because the time coefficients were made orthogonal, any effect of a fixed variable (e.g., F0, L1) is to be interpreted on the averaged differential fixations over time (Mirman, 2014).

**Table 3:** Growth curve analyses on the difference between listeners’ target and competitor fixations in the within-AP condition separately for French, English, and Korean listeners.

Group	Variable	Estimate (SE)	*t*
French	(intercept)	-0.075	-1.891
	
	Time		
	Linear	1.102	2.426
	Quadratic	2.559	7.888^∗∗∗^
	Cubic	0.551	2.056
	
	F0	0.075	12.289^∗∗∗^
	
	Time × F0		
	Linear	0.538	6.407^∗∗∗^
	Quadratic	-0.714	-8.515^∗∗∗^
	Cubic	-0.694	-8.267^∗∗∗^

English	(Intercept)	-0.152	-3.574^∗∗^
	
	Time	
	Linear	-1.619	-3.695^∗∗^
	Quadratic	0.795	2.538
	Cubic	-0.434	-1.688
	
	F0	0.016	2.144
	
	Time × F0		
	Linear	1.285	12.501^∗∗∗^
	Quadratic	0.162	1.587
	Cubic	0.899	8.760^∗∗∗^

Korean	(Intercept)	-0.151	-5.682^∗∗∗^
	
	Time		
	Linear	-1.033	-2.400
	Quadratic	0.772	2.366
	Cubic	0.367	1.493
	
	F0	-0.046	-6.084^∗∗∗^
	
	Time × F0		
	Linear	0.275	2.672^∗^
	Quadratic	1.199	11.649^∗∗∗^
	Cubic	0.207	2.009


*α = 0.0167; ^∗^ = *p* < 0.0167; ^∗∗^ = *p* < 0.003; ^∗∗∗^ = *p* < 0.0003. French model: *n* = 25; 9,188 observations. English model: *n* = 16; 5,887; observations. Korean model: *n* = 16; 6,013 observations.*

#### French Listeners

For the GCA on French listeners’ data, the significant positive *t* value for the quadratic time coefficient indicates that French listeners’ differential fixation line in the condition without an F0 rise had a convex shape. The significant positive *t* value for F0 means that French listeners had a higher differential proportion of fixations in the condition with an F0 rise than in the condition without an F0 rise. Crucially, the significant positive *t* value for the interaction between F0 and the linear time coefficient indicates that French listeners had a differential fixation line that had more of an ascending slope in the condition with an F0 rise than in the condition without an F0 rise. Furthermore, the significant negative *t* values for the interaction between F0 and the quadratic and cubic time coefficients mean that French listeners had a differential fixation line that was less convex and had more of a canonical ‘s’ shape in the condition with an F0 rise than in the condition without an F0 rise.

These results can be seen in the modeled differential proportions of fixations in **Figure 3**: From 500 ms *post* target-word onset, French listeners showed higher differential proportions of fixations, thus less lexical competition, in the presence of an F0 rise than in the absence of an F0 rise. The presence of an F0 rise thus modulates French listeners’ fixations later on in the word recognition process, making it easier to locate the word-final boundary and resulting in decreased lexical competition.

#### English Listeners

For the GCA on English listeners’ data, the significant negative *t* value for the intercept means that English listeners’ differential proportion of fixations in the condition without F0 rise was below 0. The significant negative *t* value for the linear time coefficient indicates that English listeners’ differential proportion of fixations in the condition without F0 rise had a descending slope. Importantly, the significant positive *t* values for the interaction between F0 and the linear and cubic time coefficients mean that English listeners’ differential fixation line had more of an ascending slope and more of a reversed ‘s’ shape in the condition with an F0 rise than in the condition without an F0 rise.

These results are illustrated in the modeled differential proportions of fixations in **Figure 3**: From the target-word onset until 300 ms, English listeners’ differential proportions of fixations in the two F0 conditions were divergent, with lower fixations in the condition with an F0 rise than in the condition without an F0 rise; at approximately 300 ms post target-word onset, the two differential fixation lines converged, and they diverged again shortly after 1,000 ms, with English listeners showing higher differential proportions of fixations in the condition with an F0 rise than in the condition without an F0 rise. The early difference between the two F0 conditions cannot be attributed to English listeners’ processing of the speech signal, in that it is present from the very beginning of the target word. The late divergence in the expected direction, however, confirms that English listeners could eventually incorporate the use of F0 cues to word-final boundaries in the segmentation of French speech (unlike the results of Tremblay et al., 2012).

#### Korean Listeners

Last but not least, for the GCA on Korean listeners’ data, the significant negative *t* value for the intercept means that Korean listeners’ differential proportion of fixations in the condition without an F0 rise was below 0. The significant negative *t* value for F0 indicates that Korean listeners had a *lower* differential proportion of fixations in the condition with an F0 rise than in the condition without an F0 rise. The effect of F0 is thus in the wrong direction. The positive *t* values for the interactions between F0 and the linear and quadratic time coefficients mean that Korean listeners’ differential fixation line was more ascending and more convex in the condition with an F0 rise than in the condition without an F0 rise.

These results can be observed in the modeled differential proportions of fixations in **Figure 3**: Korean listeners showed an effect of F0 in the wrong direction, showing a lower differential proportion of fixation (and thus more competition) in the condition with an F0 rise than in the condition without an F0 rise. This effect of F0 lasted from approximately 200 to 1,250 ms, and reversed thereafter. It is thus possible that Korean listeners were eventually able to use F0 cues to word-final boundaries in the within-AP condition of this experiment. What is clear from these results, however, is that at best they showed great difficulty in using this F0 rise.

#### L2 Listeners

Again, to ascertain whether Korean listeners differed from English listeners in their differential proportions of fixations, an additional GCA was run only on the L2 learners’ data in the within-AP condition, with the English group as baseline. The model with the best fit included all simple effects and all interactions. The results of this GCA and the interpretation of the GCA coefficients can be found in Appendix B in the **supplemental data (Table B4**). In brief, this GCA revealed a significant three-way interactions between L1, F0, and the linear, quadratic, and cubic time coefficients, indicating that English and Korean listeners differed from each other in the effect of F0 as a function of time: English listeners’ differential fixation lines for the two F0 conditions had similar quadratic shapes and ultimately diverged in favor of the condition with an F0 rise; by contrast, Korean listeners’ differential fixation lines differed in their quadratic shape and diverged earlier in favor of the condition without an F0 rise (cf. **Table 3**).

## Discussion

The present study investigated whether the learning of prosodic cues to word boundaries in speech segmentation is more difficult if the L1 and L2 have similar (though non-identical) prosodies than if they have markedly different prosodies. It did so by focusing on French, English, and Korean listeners’ use of F0 rise as a cue to word-final boundaries in French. French and Korean pattern similarly in that word-final boundaries in AP-final position are cued by an F0 rise; yet, they differ in that the AP-final F0 peak is aligned differently in the two languages (earlier in Korean, later in French). English differs from both French and Korean in that F0 rise signals word-initial rather than word-final boundaries. Similarity between the L1 and L2 prosodic systems was hypothesized to make the learning of F0 cues to word-final boundaries difficult. Hence, it was predicted that Korean L2 learners of French would have more difficulty in using F0 cues to word-final boundaries in French than *both* native French listeners and English L2 learners of French.

The results of the eye-tracking experiment showed that F0 cues modulated native French listeners’ differential proportions of fixations (i.e., the difference between their proportion of target fixations and their proportion of competitor fixations), with the flattening of the F0 rise resulting in a fixation line that is *lower*, more ascending, more convex, and more ‘s’-shaped than in the condition where the F0 rise was naturally present (across-AP), and with the addition of an F0 rise resulting in a fixation line that is *higher* and less convex (though also more ascending and ‘s’-shaped) than in the condition where the F0 was naturally flat (within-AP). The different directionality of the F0 effect in the across-AP and within-AP conditions and the interaction between these effects and the time coefficients provide evidence that native French listeners used the F0 rise to locate word-final boundaries in continuous French speech, and they add to the existing literature showing that prosodic cues to word-final boundaries constrain lexical access in native French listeners (e.g., Christophe et al., 2004; Michelas and D’Imperio, 2010; Tremblay et al., 2012).

The eye-tracking results also revealed that English L2 learners of French showed evidence of *ultimately* integrating F0 cues in the word recognition process, with the flattening of the F0 rise resulting in a fixation line that was *lower*, more descending, and more concave than in the condition where the F0 rise was naturally present (across-AP). Unlike native French listeners, native English listeners did not show an overall effect of F0 in their fixations in the within-AP condition; however, F0 cues modulated the shape of their differential fixations, with the addition of an F0 rise resulting in a fixation line that was more ascending, and more reversed-‘s’-shaped than in the condition where the F0 was naturally flat (within-AP), and with fixations ultimately being numerically higher in the condition with an F0 rise than in the condition without an F0 rise. These results are novel, in that they suggest that English L2 learners of French can, in fact, use F0 rise as a cue to word-final boundaries in French; as far as we know, this study is the first to report such findings. Since the English listeners in this study were somewhat less proficient than the high-proficiency English listeners in Tremblay et al. (2012) (who did not show any effect of F0), the divergent findings between the studies are likely due to the different methodologies employed in the two studies, with eye tracking providing a precise window into the time course of lexical processing and thus capturing the use of cues that may otherwise have a weaker effect in a word-monitoring task.

Finally, the results of the eye-tracking experiment showed that Korean L2 learners of French either did not use the F0 cues in the speech signal (across-AP condition) or did so but in the wrong direction (within-AP condition): The flattening of the F0 rise resulted in a fixation line that was *lower* than in the condition where the F0 rise was naturally present (across-AP), but this effect of F0 did not change as a function of time, and as such, cannot be attributed to the speech signal;^8^ and the addition of an F0 rise resulted in a fixation line that was *lower* (not higher), more ascending, and more convex than in the condition where the F0 was naturally flat (within-AP). Since the directionality of the F0 effect numerically reverses toward the end of the word recognition process, it is possible that Korean L2 learners of French eventually become able to integrate F0 cues in a target-like manner in their speech segmentation. Overall, however, their pattern of results suggests that they experience great difficulty using F0 rise as a cue to word-final boundaries in French, a finding that is also novel.

These results suggest that the similarity between the L1–L2 prosodic systems in the use of F0 cues makes the learning of L2 segmentation cues difficult for L2 learners, in line with the proposed Prosodic-Learning Interference Hypothesis.^9^ We suspect that Korean listeners’ difficulty using the F0 rise as a cue to word-final boundaries in French stems from the different alignments of the AP-final F0 rise in French and in Korean. When hearing an F0 rise in French, Korean L2 learners of French must adjust the timing with which they anticipate a phrase-final (thus, word-final) boundary. If Korean listeners parse French the way they parse Korean, they might wait until the F0 begins lowering to anticipate a word-final boundary; at that point in time, it will already be too late for them to make use of this F0 information in French, as the next word will have already begun. This may explain why Korean listeners had difficulty using the F0 rise in French. If anything, the results in the within-AP condition suggested that Korean listeners initially interpreted this F0 rise as signaling a *word-initial* boundary in French. The late alignment of the F0 rise may thus have been perceived by Korean listeners as being located on the first syllable of the adjective following the monosyllabic word (e.g., *chat lé–*), thus resulting in more lexical competition from the disyllabic word (e.g., *chalet*) in the presence of an F0 rise than in the absence of such a rise.

We believe that the prosodic similarity between French and Korean poses a learnability problem for Korean L2 learners of French and in turn results in speech segmentation difficulties. From the effect of L1 on processing alone, English L2 learners of French should have *more* difficulty in using F0 rise to locate word-final boundaries in French than Korean L2 learners of French, as F0 rise signals *word-initial* rather than phrase-final boundaries in English. Yet, English L2 learners of French were ultimately able to integrate F0 cues to word-final boundaries in a target-like manner to segment French speech, both in non-AP-final (within-AP condition) and AP-final (across-AP condition) positions. Since our L2 groups were matched in both their French proficiency and French experience, the observed difference between the two L2 groups suggest that the prosodic similarity between French and Korean may pose a learnability problem for Korean listeners, consistent with the Prosodic-Learning Interference Hypothesis.

Similarity between L1-L2 prosodic systems may hurt L2 learning for two reasons: L2 listeners may perceptually assimilate L2 prosodic cues to L1 prosodic cues and/or they may not experience parsing failure as a result of not using L2 prosodic cues. First, Korean L2 learners of French may perceive the F0 rise in French as similar to that in Korean. As a result, they may not readjust their use of segmentation cues. Similar perceptual assimilations have been reported for the perception of segments (for a discussion of PAM-L2 and SLM, see Best and Tyler, 2007 and Flege, 1995, respectively). However, since F0 cues are unlikely to be perceived categorically (at least in French and Korean), the exact process underlying the assimilation of F0 cues in the L1 and L2 would likely be different from that postulated for L1 and L2 segments. Second, L2 learners may not readjust their use of segmentation cues if these unadjusted cues do not cause parsing failure (i.e., if they do not result in the greater activation of L2 competitor words over L2 target words; for such a proposal, see Carroll, 2004). The present results do not adjudicate between these two types of mechanisms, but they pave the way for further research to try to tease them apart.

The main contribution of this study is in demonstrating that learning to segment speech in the L2 is difficult if a particular prosodic cue signals the same word boundary in the L1 and L2 but does so differently. We have provided evidence that Korean L2 learners of French, unlike English L2 learners of French matched to them in French proficiency and French experience, have great difficulty learning to use F0 rise to locate word-final boundaries in French, a result which we hypothesize is due to the different alignments of the AP-final F0 peak in Korean (earlier) and French (later). To the best of our knowledge, this study is the first to report that similarity between the L1 and L2 can hurt L2 learning in the domain of sentential prosody. Further research should examine how differences in this AP-final F0-peak alignment impact Korean listeners’ segmentation of French speech. Our findings also raise the question of whether L2 learning is similarly impacted by subtle prosodic differences that manifest themselves differently (e.g., cues that have different alignments vs. different strengths) or that are used to express a categorical distinction in one language but not the other. Answering these questions would make an important contribution to the understanding of how non-native listeners become (or do not become) able to segment speech successfully in an L2.

The findings of this study also raise questions about the mechanisms underlying L2 learners’ encoding of prosodic cues. Prosodic information in French is, at least to a large degree, independent from segmental information: The same words can be realized very differently depending on their position in the AP. This makes it unlikely that native French listeners would encode the prosody of each exemplar French word they hear in their lexical representations. Computing the prosody of the utterance independently of its segmental content, with listeners aligning words with the prosodic constituents of the utterance, may be a more efficient strategy. Korean L2 learners of French, for whom prominence is also phrasal, may also compute the prosody of the French utterance somewhat (but perhaps not completely) independently of its segmental content (since the tonal pattern of the AP in Korean is partly influenced by the AP-initial segment), but with the alignment of the prosodic constituents (signaled by prosodic cues such as F0 rise) being slightly off and thus resulting in speech segmentation difficulties. In contrast, English L2 learners of French, for whom prominence is both lexical and phrasal, may begin the learning of French words by encoding a great deal of prosodic details for each exemplar, and only later become less reliant on such lexical encoding. Importantly, even if English listeners were to adopt a different strategy from French listeners at their onset of learning French, ultimately they showed the right pattern of fixations when segmenting French, albeit late in the word recognition process. Further research should shed light on the precise mechanisms that underlie listeners’ encoding of prosodic information, whether this encoding varies across languages, and if so, how these differences affect the L2 learning of prosody.

## Author Contributions

AT: Stimuli creation, experimental design, data collection, data analysis, writing of manuscript. MB: Data analysis, writing of manuscript. CC: Stimuli creation, experimental design, data collection, data analysis, writing of manuscript. JC: Data collection, writing of manuscript.

## Conflict of Interest Statement

The authors declare that the research was conducted in the absence of any commercial or financial relationships that could be construed as a potential conflict of interest.

## Footnotes

1 Existing acoustic metrics of rhythm such as the normalized Pairwise Variability Index (*nPVI*; Low et al., 2000) and the proportion of speech time dedicated to vocalic intervals *(%V*; Ramus et al., 1999) have not compared these languages, and even if they had, inconsistencies in how these metrics map different languages in a rhythmic space (e.g., Grabe and Low, 2002 vs. Ramus, 2002) would likely make it difficult to draw firm conclusions on the degree of similarity between the above language pairs. The fact that these metrics additionally reflect syllable structure differences among languages could also make these comparisons difficult (e.g., Korean and French have different syllable structures).

2 We do not seek to claim that F0 plays a more important role than other prosodic cues (e.g., duration) in speech segmentation (in French or across languages). Ultimately, F0 is only one of the prosodic cues that contribute to signaling word boundaries, and it is only one of the cues through which the prosodic system of the language is realized. We focus on F0 because it provides an easier and clearer test of the hypothesis that the learning of L2 segmentation cues is shaped by the degree of similarity between the L1 and the L2 (particularly for the language pairs selected for this study).

3 F0 rise does not cue word-final boundaries within phrases in French. Throughout the paper, we will refer to the use of F0 cues to word-final boundaries in French with the understanding that such cues occur in phrase-final position and thus signal word-final boundaries only in phrase-final position.

4 They did if the following word had atypical prosody (e.g., HL) but not if it had prototypical prosody (e.g., LH). The authors attribute the asymmetrical effect of lengthening to a segmental confound in their design.

5 Among the Korean listeners, 2 were native speakers of the Chungcheong dialect, 2 were native speakers of the Kyungsang dialect, and 1 was a native speaker of the Gangwon dialect; all other Korean listeners were native speakers of the Seoul dialect. Kyungsang Korean differs from Seoul Korean in that it has lexical pitch accents, with some accents ending with an L tone rather than an H tone (e.g., Lee and Zhang, 2014). However, recent research suggests that young Kyungsang Korean speakers are in the process of losing these different pitch accents, possibly due to the close contact with and influence of Seoul Korean (Lee and Jongman, 2015). To ensure that our two Kyungsang Korean speakers did not drive any of the results, we ran our statistical analyses (presented further below) both with and without these two speakers. The variables that reached significance were exactly the same in the two different analyses. We therefore kept these two speakers in our analyses.

6 More Korean and English L2 learners of French were tested, but a subset of each group was selected so that they would match in both their proficiency in and experience with French.

7 Given the complexity of this analysis and the much larger dataset it runs on (as compared to those used in typical mixed-effect models), adding additional random variables to the analysis requires significant computing power, with each analysis taking several hours to run. For this reason, in the present study, only participant is used as random intercept. The above fixed variables are not added as random slopes, because the analyses often fail to converge.

8 It is unclear why this effect of F0 emerges since it cannot be attributed to the speech signal.

9 One concern that might be raised with this study is the fact that the target and competitor words in the eye-tracking experiment were presented orthographically, and Korean uses a different orthographic system from French and English, thus possibly making the task more difficult for Korean L2 learners of French. This concern is unlikely to explain the present results, however. First, Korean speakers learn the roman alphabet from the age of 6 when they begin learning English. Second, the participants had 4,000 ms to preview the orthographic words before they heard the target word. This should have given them plenty of time to orthographically decode the words on the screen. Third, the dependent variable in the statistical analysis was the difference between the proportions of target fixations and the proportions of competitor fixations. This difference closely reflects the amount of lexical activation of both the target and competitor words, and thus factors out overall speed differences due to the decoding of the orthographic words (i.e., such speed differences would affect fixations to both target and competitor words, not one over the other). The use of orthography in this experiment is thus very unlikely to explain why Korean listeners had more difficulty than English listeners in using the F0 rise as a cue to word-final boundaries in French.
