# Supplementary material for: Effects of the Native Language on the Learning of Fundamental Frequency in Second-Language Speech Segmentation
Source: Front Psychol. 2016 Jun 29;7:985. doi: 10.3389/fpsyg.2016.00985 (PMC4925665; doi:10.3389/fpsyg.2016.00985)
Supplement: Supplementary file 1 [file Data_Sheet_1.DOCX]

**Appendix A**


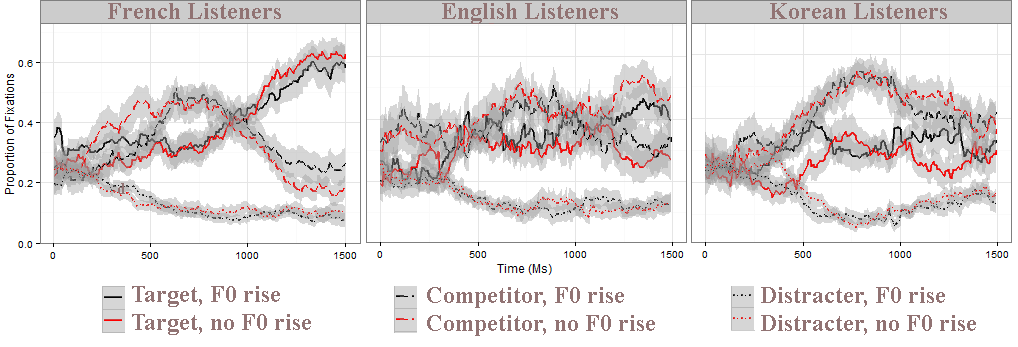


**Figure A1.** Participants’ proportions of fixations in the across-AP condition


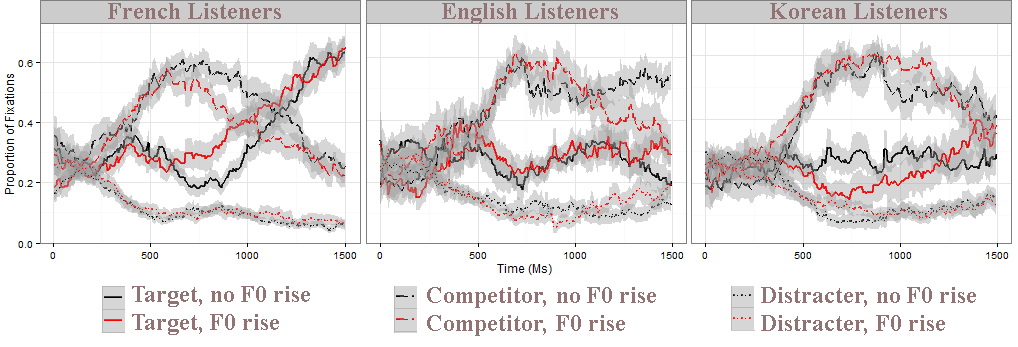


**Figure A2.** Participants’ proportions of fixations in the within-AP condition

**Appendix B.**

**Table B1.** Growth curve analysis on the difference between listeners’ target and competitor fixations in the across-AP condition, including French, English, and Korean listeners, with the French group as baseline

| Variable | Estimate (SE) | *t* |  |
| --- | --- | --- | --- |
| (intercept) | 0.886 | 2.718 | * |
| Time |  |  |  |
| Linear | 1.769 | 1.903 |  |
| Quadratic | –0.166 | 4.953 | *** |
| Cubic | –0.074 | <\|1\| |  |
| L1 (English) | –0.185 | –1.430 |  |
| L1 (Korean) | –0.015 | –3.578 | *** |
| F0 | 0.886 | –2.545 | * |
| Time × L1 (English) |  |  |  |
| Linear | –0.447 | <\|1\| |  |
| Quadratic | –1.217 | –2.120 | * |
| Cubic | 0.311 | <\|1\| |  |
| Time × L1 (Korean) |  |  |  |
| Linear | –1.167 | –1.572 |  |
| Quadratic | –1.236 | –2.170 | * |
| Cubic | 0.229 | <\|1\| |  |
| Time × F0 |  |  |  |
| Linear | 1.253 | 14.989 | *** |
| Quadratic | 0.203 | 2.440 | * |
| Cubic | –0.496 | –5.948 | *** |
| F0 × L1 (English) | –0.085 | –8.715 | *** |
| F0 × L1 (Korean) | –0.036 | –3.675 | *** |
| Time × F0 × L1 (English) |  |  |  |
| Linear | –2.745 | –20.469 | *** |
| Quadratic | –0.747 | –5.585 | *** |
| Cubic | 0.362 | 2.696 | ** |
| Time × F0 × L1 (Korean) |  |  |  |
| Linear | –1.398 | –10.539 | *** |
| Quadratic | –0.092 | <\|1\| |  |
| Cubic | 0.571 | 4.303 | *** |

*Note.* α = .05, * = *p*<.05, ** = *p*<.01, *** = *p*<.001; *n*=57, 21,081 observations

The baseline in this analysis is the difference between French listeners’ proportions of target and competitor fixations in the natural condition where the target word ends with an F0 rise. Because the time coefficients were made orthogonal, any effect of a fixed variable (e.g., F0, L1) is to be interpreted on the averaged differential fixations over time (Mirman, 2014).

The significant positive *t* value for the intercept indicates that French listeners’ differential proportion of fixations in the condition with an F0 rise was above 0. The significant positive *t* value for the quadratic time coefficient means that French listeners’ differential fixation line in the condition with an F0 rise had a convex (i.e., U) shape. The significant negative *t* value for L1 (Korean) indicates that Korean listeners had a lower differential proportion of fixations than French listeners in the condition with an F0 rise. The significant negative *t* value for F0 means that French listeners had a lower differential proportion of fixations in the condition without an F0 rise than the condition with an F0 rise. The significant negative *t* values for the interaction between L1 and the quadratic time coefficient (English and Korean) indicate that English and Korean listeners had differential fixation lines that were less convex than French listeners in the condition with an F0 rise. The significant positive *t* values for the interaction between F0 and the linear and quadratic time coefficients mean that French listeners had a more ascending slope and more convex differential fixation line in the condition without an F0 rise than the condition with an F0 rise. The significant negative *t* value for the interaction between F0 and the cubic time coefficient indicates that French listeners’ differential fixation line had more of a canonical ‘s’ shape in the condition without an F0 rise than in the condition with an F0 rise. The significant negative *t* values for the interaction between L1 and F0 (English and Korean) mean that the effect of F0 decreased or reversed in the L2 groups as compared to the French group. Finally, and importantly, the three-way interactions between F0, L1, and the time coefficients (linear, quadratic, and cubic for the English group, and linear and quadratic for the Korean group) indicate that English and Korean listeners differed from French listeners in the effect of F0 they showed as a function of time.

**Table B2.** Growth curve analysis on the difference between listeners’ target and competitor fixations in the across-AP condition, including English and Korean listeners, with the English group as baseline

| Variable | Estimate (SE) | *t* |  |
| --- | --- | --- | --- |
| (intercept) | 0.007 | <\|1\| |  |
| Time |  |  |  |
| Linear | 0.560 | 1.018 |  |
| Quadratic | 0.463 | 1.472 |  |
| Cubic | 0.184 | 1.053 |  |
| L1 | –0.104 | –2.030 |  |
| F0 | –0.101 | –13.160 | *** |
| Time × L1 |  |  |  |
| Linear | –0.842 | –1.087 |  |
| Quadratic | 0.070 | <\|1\| |  |
| Cubic | –0.120 | <\|1\| |  |
| Time × F0 |  |  |  |
| Linear | –1.492 | –14.222 | *** |
| Quadratic | –0.543 | –5.182 | *** |
| Cubic | –0.136 | –1.295 |  |
| F0 × L1 | 0.050 | 4.631 | *** |
| Time × F0 × L1 |  |  |  |
| Linear | 1.346 | 9.156 | *** |
| Quadratic | 0.653 | 4.445 | *** |
| Cubic | 0.210 | 1.428 |  |

*Note.* α = .05; * = *p*<.05; ** = *p*<.01; *** = *p*<.001; *n*=32, 11,845 observations

The baseline in this analysis is the difference between English listeners’ proportions of target and competitor fixations in the natural condition where the target word ends with an F0 rise. Because the time coefficients were made orthogonal, any effect of a fixed variable (e.g., F0, L1) is to be interpreted on the averaged differential fixations over time (Mirman, 2014).

The significant negative *t* value for F0 means that English listeners had a lower differential proportion of fixations in the condition without an F0 rise than in the condition with an F0 rise. The significant negative *t* value for the interactions between F0 and the linear and quadratic coefficients indicate that English listeners’ differential fixation line had more of a descending slope and concave shape in the condition without an F0 rise than in the condition with an F0 rise. The significant positive *t* value for the interaction between F0 and L1 means that English listeners’ differential proportions of fixations indicates that the reduction in F0 in the condition without an F0 rise was greater for Korean listeners than for English listeners. Finally, and importantly, the three-way interactions between F0, L1, and the linear and quadratic time coefficients indicate that Korean listeners differed from English listeners in the effect of F0 they showed as a function of time.

**Table B3**. Growth curve analysis on the difference between listeners’ target and competitor fixations in the within-AP condition, including French, English, and Korean listeners, with the French group as baseline

| Variable | Estimate (SE) | *t* |  |
| --- | --- | --- | --- |
| (intercept) | –0.078 | –2.337 | * |
| Time |  |  |  |
| Linear | 1.156 | 3.013 | ** |
| Quadratic | 2.525 | 8.965 | *** |
| Cubic | 0.561 | 2.386 | * |
| L1 (English) | –0.075 | –1.403 |  |
| L1 (Korean) | –0.073 | –1.369 |  |
| F0 | 0.075 | 12.341 | *** |
| Time × L1 (English) |  |  |  |
| Linear | –2.765 | –4.505 | *** |
| Quadratic | –1.738 | –3.855 | *** |
| Cubic | –0.991 | –2.633 | * |
| Time × L1 (Korean) |  |  |  |
| Linear | –2.189 | –3.580 | *** |
| Quadratic | –1.753 | –3.902 | *** |
| Cubic | –0.195 | <\|1\| |  |
| Time × F0 |  |  |  |
| Linear | 0.538 | 6.448 | *** |
| Quadratic | –0.715 | –8.566 | *** |
| Cubic | –0.693 | –8.297 | *** |
| F0 × L1 (English) | –0.059 | –6.059 | *** |
| F0 × L1 (Korean) | –0.120 | –12.518 | *** |
| Time × F0 × L1 (English) |  |  |  |
| Linear | 0.747 | 5.596 | *** |
| Quadratic | 0.878 | 6.594 | *** |
| Cubic | 1.593 | 11.942 | *** |
| Time × F0 × L1 (Korean) |  |  |  |
| Linear | –0.263 | –1.991 | * |
| Quadratic | 1.914 | 14.480 | *** |
| Cubic | 0.900 | 6.806 | *** |

*Note.* α = .05, * = *p*<.05, ** = *p*<.01, *** = *p*<.001; *n*=57, 21,088 observations

The baseline in this analysis is the difference between French listeners’ proportions of target and competitor fixations in the natural condition where the target word does not have an F0 rise. Because the time coefficients were made orthogonal, any effect of a fixed variable is to be interpreted on the average differential fixations over time (Mirman, 2014).

The significant negative *t* value for the intercept indicates that French listeners’ differential proportion of fixations in the condition without an F0 rise was below 0. The significant positive *t* values for the linear, quadratic, and cubic time coefficients means that French listeners’ differential fixation line in the condition without an F0 rise had an ascending slope, convex shape, and reversed ‘s’ shape. The significant negative *t* values for L1 (English and Korean) indicates that English and Korean listeners had lower differential proportions of fixations than French listeners in the condition without an F0 rise. The significant positive *t* value for F0 means that French listeners had a higher differential proportion of fixations in the condition with an F0 rise than in the condition without an F0 rise. The significant negative *t* values for the interactions between L1 and the linear and quadratic time coefficients (English and Korean) indicate that English and Korean listeners had differential fixation lines that had a less of an ascending slope and less convex shape than French listeners in the condition without an F0 rise, and the significant negative *t* value for the same interaction but with the cubic time coefficient (English) means that English listeners had a differential fixation line that had less of a reversed ‘s’-shape than French listeners. The significant positive *t* value for the interaction between F0 and the linear time coefficient means that French listeners’ differential fixation lines had more of an ascending slope in the condition with an F0 rise than in the condition without an F0 rise. The significant negative *t* values for the interactions between F0 and the quadratic and cubic time coefficients indicates that French listeners’ differential fixation line was less convex and had less of a reversed ‘s’ shape in the condition with an F0 rise than in the condition without an F0 rise. The significant negative *t* values for the interaction between L1 and F0 (English and Korean) mean that the effect of F0 decreased or reversed in the L2 groups as compared to the French group. Finally, and importantly, the three-way interactions between F0, L1, and the time coefficients (linear, quadratic, and cubic for the English group, and linear and cubic for the Korean group) indicate that English and Korean listeners differ from French listeners in the effect of F0 they show as a function of time.

**Table B4.** Growth curve analysis on the difference between listeners’ target and competitor fixations in the within-AP condition, including English and Korean listeners, with the English group as baseline

| Variable | Estimate (SE) | *t* |  |
| --- | --- | --- | --- |
| (intercept) | –0.153 | –4.293 | *** |
| Time |  |  |  |
| Linear | –1.600 | –3.698 | *** |
| Quadratic | 0.779 | 2.434 | * |
| Cubic | –0.426 | –1.694 |  |
| L1 | 0.002 | <\|1\| |  |
| F0 | 0.016 | 2.129 | * |
| Time × L1 |  |  |  |
| Linear | 0.566 | <\|1\| |  |
| Quadratic | –0.007 | <\|1\| |  |
| Cubic | 0.792 | 2.232 | * |
| Time × F0 |  |  |  |
| Linear | 1.285 | 12.391 | *** |
| Quadratic | 0.162 | 1.572 |  |
| Cubic | 0.900 | 8.690 | *** |
| F0 × L1 | –0.062 | –5.827 | *** |
| Time × F0 × L1 |  |  |  |
| Linear | –1.010 | –6.940 | *** |
| Quadratic | 1.037 | 7.140 | *** |
| Cubic | –0.693 | –4.764 | *** |

*Note.* α = .05, * = *p*<.05, ** = *p*<.01, *** = *p*<.001; *n*=32, 11,900 observations

The baseline in this analysis is the difference between English listeners’ proportions of target and competitor fixations in the natural condition where the target word does not have an F0 rise. Because the time coefficients were made orthogonal, any effect of a fixed variable (e.g., F0, L1) is to be interpreted on the averaged differential fixations over time (Mirman, 2014).

The significant negative *t* value for the intercept indicates that English listeners’ differential proportion of fixations in the condition without an F0 rise was below 0. The significant negative *t* value for the linear time coefficient and the significant positive *t* value for the quadratic time coefficient mean that English listeners’ differential fixation line in the condition without an F0 rise had a descending and convex shape. The significant positive *t* value for F0 indicates that English listeners had a higher differential proportion of fixations in the condition with an F0 rise than in the condition without an F0 rise. The significant positive *t* value for the interaction between L1 and the cubic time coefficient means that Korean listeners’ differential fixation line in the condition without an F0 rise had more of a reversed ‘s’ shape than English listeners’ corresponding differential line. The significant positive *t* values for the interaction between F0 and the linear and cubic time coefficients indicate that English listeners’ differential fixation line had more of an ascending and reversed ‘s’-shape in the condition with an F0 rise than in the condition without an F0 rise. The negative *t* value for the interaction between F0 and L1 means that Korean listeners showed a weaker or reversed effect of F0 as compared to English listeners. Finally, and importantly, the three-way interactions between F0, L1, and the linear, quadratic, and cubic time coefficients indicate that Korean listeners differed from English listeners in the effect of F0 they showed as a function of time.
